# Prevalence and Associated Factors of Hypertension: A Crossectional Community Based Study in Northwest Ethiopia

**DOI:** 10.1371/journal.pone.0125210

**Published:** 2015-04-24

**Authors:** Solomon Mekonnen Abebe, Yemane Berhane, Alemayehu Worku, Assefa Getachew

**Affiliations:** 1 Institute of Public health, College of Medicine and Health Sciences, University of Gondar, Gondar, Ethiopia; 2 Addis Continental Institute of Public Health, Addis Ababa, Ethiopia; 3 School of Public Health, Addis Ababa University, Addis Ababa, Ethiopia; 4 School of Medicine, College of Medicine and Health Sciences, University of Gondar, Gondar, Ethiopia; Shanghai Institute of Hypertension, CHINA

## Abstract

**Background:**

Hypertension, being the root cause of many of the body sytem and organs failure, remains to be a major public health challenge globally. Though the problem is huge in both developed and developing countries, data are scarce in developing countries like Ethiopia. Therefore, this study was aimed to determine the magnitude and associated factors of hypertension in North West Ethiopia.

**Methods:**

A cross-sectional survey was conducted on adults aged 35 years and above in the rural and urban communities of Dabat district and Gondar town in 2012. The data were collected using the WHO STEPwise strategy. Hypertension was defined as having a Systolic blood pressure of ≥140 mmHg and/ or a Diastolic BP of ≥ 90mmHg or a reported use of anti-hypertensive medications for raised blood pressure. Prevalence was computed with a 95% confidence interval. Selected risk factors were assessed using a biviarete logistic regression.

**Results:**

A total of 2200 participants were included in the study. The median age (±SD) was 47 (±12.4) years. The overall prevalence of hypertension was found to be 27.9% [95% CI 26.0, 29.8], with the proportion in the urban and rural residents being 30.7% and 25.3% respectively. The prevalence of hypertension was 29.3% for women and 26.3% for men. Out of the 598 hypertensive patients 241 (40.3%) had blood pressure measurements, and 99 (16.6%) had known hypertension and were on treatment. The proportion of systolic and diastolic hypertension in this subgroup of adults was 133(6.2%). The multivariable logistic regression analysis showed older age (AOR = 1.06; 1.05, 1.07), raised fasting glucose (AOR = 1.01; 1.001, 1.01), alcohol consumption (AOR = 1.71; 1.24, 2.36), and raised BMI (AOR =1.07; 1.04, 1.10) were significantly associated with hypertension.

**Conclusion:**

The prevalence of hypertension was considerably higher in rural areas than previously reported. The health system needs to develop strategies to increase the reach of relevant screening and diagnostic services to both rural and urban populations.

## Introduction

Hypertension is a worldwide public-health challenge and a leading modifiable risk factor for cardiovascular disease (CVD) and death. According to the WHO Global Health Observary Report, globally, the overall prevalence of Hypertension in adults aged 25 and over was around 40% in 2008 and was is estimated to cause 7.5 million deaths, about 12.8% of the total of all deaths worldwide. Globally the number of people with uncontrolled hypertension rose by 70% between 1980 and 2008. The rising epidemic of hypertension is thought to be due to mechanization, population growth and ageing [1],[2].

There has been a wide range of prevalence of hypertension around the world, with the lowest prevalence in rural India (3.4% in men and 6.8% in women) and the highest prevalence (78%) for South Africa and Poland (68.9% in men and 72.5% in women). Similarly the prevalence of hypertension is rising among older adults in Sub-Saharan Africa [3,4].

Current disease estimates for Sub-Saharan Africa (SSA) are based on sparse data, but projections indicate increases in non-communicable diseases (NCDs) caused by demographic and epidemiologic transitions; however, hypertension control assumes a relatively low priority and little experience exists in implementing sustainable and successful programs. There is a wide disparity (0.4 to 43%) in the prevalence of hypertension and obesity in Sub-Saharan Africa [5].

A study conducted in Ethiopia in the last decade showed that the prevalence of CVD risk factor increased rapidly [6]. A study done on Gondar city of Ethiopia showed a 28.3% prevalence of hypertension [7]. A similar study done on urban Commercial Bank employees in Addis Ababa, Ethiopia, showed a 19.1% prevalence of hypertension which indicated a wide disparity in magnitude due to the significance of lifestyle for hypertension etiology [8].

The prevention and control of hypertension has not received due attention in Ethiopia compared with other computing diseases (HIV/AIDS, tuberculosis, and malaria). Recent evidences indicate that hypertension and raised blood sugar are increasing [9]. However, as most of the studies in our region are institution- based or urban- focused, very few studies have emphasized rural population such as Butajira DHSS earlier than 8 years, (8.2% of women and 12.3%) [10]. Documented prevalence of hypertension at rural community level has been limited, particularly in the study area. Thus, this study was intended to assess the prevalence of Hypertension and associated factors among rural and urban populations in northwest Ethiopia.

## Methods

### Study Areas and Population

#### Study Areas

This study was conducted in Gondar town and Dabat rural kebeles of North Gondar, which were parts of a larger research project on the epidemiology of diabetes mellitus in northwest Ethiopia. Gondar is located 727 km northwest of the capital city of Ethiopia, Addis Ababa. It is a densely populated historical city of the region with an estimated urban population of 254,420, 120,569 male and 133,851 female, with an average household size of 4.0 in 2013 [11]. The rural district of Dabat has an estimated population of 44,723 largely living on subsistence farming [12]. The study utilized a cross-sectional community-based design. All permanent residents in the study area who were 35 years and older were eligible to participate in the study.

A multistage cluster random sampling strategy was used to select study participants from urban and rural locations. First, clusters (smallest administrative units, known as *kebeles* in Ethiopia) were selected using simple random sampling after obtaining the latest list from the district administration. Secondly, households were selected within each cluster using the systematic random sampling technique. Finally, one individual was selected out of eligible adults in each household using simple random sampling. Details of the study population and sampling procedures were previously described [13].

#### Data collection

Data collection and training were conducted in accordance with the WHO Stepwise approach recommended for non-communicable disease surveillance [14]. The WHO and IDA criterion was used to classify hypertension with systolic blood pressure (SBP) of ≥140mmHg and /or a diastolic blood pressure (DBP) ≥ 90 mmHg or known hypertensive patients on treatment. Anthropometric measurements were taken using standardized techniques and calibrated equipment. Blood pressure (BP) was measured using a digital measuring device (Mars MS-700AMI, China) with participants sitting after resting for at least five minutes. Three BP measurements were taken with at least three-minute intervals between the consecutive measurements. In accordance with the WHO recommendation, the mean systolic and diastolic BP from the second and third measurements was considered for analyses.

Impaired fasting blood glucose levels (IFG) were between 110 and 125 mg/dl. Waist circumference (WC) was categorized as low risk if it was less than 94 cm for men, and less than 80 cm for women; high risk if it was 94 cm or more for men, and 80 cm or more for women [14–16]. Body mass index (BMI) was calculated as the ratio of weight in kilograms to the square of height in meters. BMI was used to define underweight (BMI < 18.5), normal (18.5 ≤ BMI < 25.0), overweight (25.0 ≤ BMI < 30.0), and obese (BMI ≥ 30) adults. Current alcohol consumption was assessed by asking participants to respond by ticking “Yes /No” to the question, “have you consumed any alcoholic drink, such as beer, wine, tela, tej, local areki, fermented cider in the last 30 days?”. Data about current smoking was found out by asking participants to respond in the same to the question, “Do you currently smoke any tobacco products, such as cigarettes?” Moderate physical activity was considered as “Yes” for those participants who walked at least for 10 minutes continuously on a daily basis.

Data were collected by trained field workers that included local community enumerators, laboratory technicians, and nurses by going house-to-house. To ensure the quality of the interview, data collectors were trained by the principal investigator, and later on random checks were carried out by field supervisors and the principal investigator.

#### Data analysis

Double data entry procedures were done using the EPI Info statistical software [13]. A stratified analysis was also performed to see residence and sex specific proportion. Hypertension having a systolic blood pressure (SBP) ≥140mmHg and /or diastolic blood pressure (DBP) ≥ 90 mmHg or known hypertensive patients on treatment was used to diagnose hypertension which was confirmed by repeating the measurement three times and taking the average. Isolated diastolic hypertension (IDH) having a (systolic blood pressure < 140 mmHg and diastolic blood pressure ≥ 90 mmHg) and Isolated systolic hypertension (ISH) having a (systolic blood pressure ≥ 140 mmHg and diastolic blood pressure <90 mmHg) was used to diagnose IDH and ISH respactivly. Logistic regression was applied to identify the risk factors for hypertension. Initially, potential risk factors were evaluated using bivariate analyses; an arbitrary p-value of < 0.20 was used as criteria to include it in the multivariable logistic regression model to control confounding effects, and the results were considered statistically significant at P-value ≤ 0.05. The independent variables, like socio-demographic factors and health related life-style characteristics (engaging in physical activity, Body mass Index, abdominal obesity, dietary habits, instance of alcoholic drink, and smoking) of the study population were computed in the multivariable logistic regression analysis. The value 0 was given to a normotensive case (a person having systolic blood pressure (SBP) <140mmHg and /or diastolic blood pressure (DBP) < 90 mmHg or no previous history of hypertension, and 1 to a hypertensive case (a person having systolic blood pressure (SBP) ≥140mmHg and /or a diastolic blood pressure (DBP) ≥ 90 mmHg or known hypertensive patients on treatment was used to make the outcome classified. Statistical analysis was performed using STATA version 12 software.

#### Ethical Statement

Subjects who volunteered to participate in the study were included after signing a written agreement. Identified cases for hypertension were referred to the nearby clinic for further treatment and follow-up. The protocol and the written consent was approved by the Institution Review Board (IRB) of the University of Gondar.

## Results

### Socio-demographic characteristics of the study population

A total of 2200 subjects were initially invited for the survey, but 59 refused to participate, resulting in a response rate of 97.3%. Of this, about 1050 (49%) were urban dwellers and 53.7% were women. The median age (±SD) of the study group was 47.0 (±12.4) years. The Mean (±SD) systolic blood pressure (mmHg) was 129.4 mmHg (16.9) in urban and 129.5 mmHg (16.6) in rural dwellers. The Mean (±SD) diastolic Blood Pressure was higher among urban men 80.4 mmHg (10.3) than rural men, 77.5 mmHg (9.4). The difference was not significant in woman. The mean age for subjects with hypertension was 56.2 years; the mean age was 56.8.years for rural residents and 55.6 years for urban dwellers; 55.7years for women and 56.8 years for men Table 1. The socio-demographic characteristics of the study population were presented in the previous study [13]]. The mean systolic and diastolic blood pressure and the health related life style characteristics of the study population were presented in Table 2.

**Table 1:** Socio-demographic characteristics of the study population by residence and sex for adults age 35 and above years, North West Ethiopia, 2012.

Variable	Study participant			
	Urban		Rural	
**Age Group (years)**	**Male n (%)**	**Female n (%)**	**Male n (%)**	**Female n (%)**
35–44	142 (40.5)	265 (37.9)	280 (44.4)	215 (46.6)
45–54	99 (28.2)	201 (28.8)	159 (25.2)	107 (23.2)
55–64	39 (11.1)	139 (19.9)	103 (16.4)	84 (18.2)
≥ 65	71 (20.2)	94 (13.5)	88 (13.9)	55 (11.9)
**Religion**				
Orthodox	311 (89.9)	654 (94.5)	624 (99.1)	455 (98.7)
Muslim	32 (9.2)	36 (5.2)	6 (0.9)	6 (1.3)
Others	3 (0.9)	2 (0.3)	0 (0.00)	0 (0.00)
**Level of Education**				
Non Formal school.	137 (39)	438 (62.7)	544 (86.4)	437 (62.7)
Grade 1–6	23 (6.6)	50 (7.2)	75 (11.9)	20 (4.3)
Grade 7–12	102 (29.1)	138 (19.7)	9 (1.4)	3 (0.65)
Diploma and above	68 (19.4)	20 (2.9)	1 (0.2)	0 (0.00)
Refused	21 (5.9)	53 (7.6)	1 (0.2)	1 (0.22)
**Marital status**				
Never married	36 (10.3)	49 (7.02)	5 (0.8)	0 (0.00)
Currently married	269 (77.1)	412 (59)	608 (96.5)	278 (60.3)
Separated	18 (5.2)	47 (6.7)	1 (0.2)	5 (1.1)
Divorced	9 (2.6)	60 (8.6)	6 (0.95)	34 (7.4)
Widowed	10 (1.6)	129 (18.5)	10 (1.6)	144 (31.2)
**BMI kg/m2**				
≤ 18	26 (7.4)	80 (11.5)	183 (29.1)	155 (33.7)
18–24	257 (73.2)	460 (66)	435 (69.1)	299 (65)
25 and above	68 (19.4)	157 (22.6)	12 (1.9)	6 (1.3)
**Family history**				
Yes	39 (5.6)	35 (10.03)	4 (0.9)	0 (0.00)
No	658 (94.4)	314 (89.97)	456 (99.1)	630 (100)
**Smoking**				
Yes	18 (5.1)	0 (0.00)	5 (0.8)	1 (0.22)
**No**	333 (94.9)	697 (100)	625 (99.2)	460 (99.8)
**Alcoholic drink within the past 30 day**				
Yes	140 (39.9)	104 (14.9)	619 (98.2)	441 (95.7)
No	211 (60.1)	595 (85.1)	11 (1.75)	20 (4.3)

**Table 2:** Prevalence hypertension, isolated systolic hypertension, isolated diastolic hypertension and systolic and diastolic hypertension by age groups and gender among residence of North West Ethiopia (2012).

Characteristics	Urban		Rural		Both
hypertension n = 598					
**Age**	**Male**	**Female**	**Male**	**Female**	
Overall	102 (29.1)	220 (31.5%)	156 (24.8%)	120 (26.0%)	598 (27.9%)
35–44	24 (16.9%)	40 (15.1%)	46 (19.4%)	25 (11.6%)	135 (14.9%)
45–54	23 (23.2%)	64 (31.8%)	28 (17.6%)	25 (23.4%)	140 (24.7%)
55–64	21 (53.8%)	65 (46.8%)	30 (29.1%)	40 (47.6%)	156 (42.7%)
≥ 65	34 (47.9%)	51 (54.3%)	52 (59.1%)	30 (54.6%)	167 (54.2%)
**Isolated systolic hypertension** n = 294					
Overall	40 (12.8%)	88 (14.2%)	93 (16.2%)	73 (16.9%)	294 (13.7%)
35–44	8 (6.2%)	13 (5.3%)	26 (9.9%)	16 (7.6%)	63 (7.4%)
45–54	5 (5.6%)	20 (11.7%)	14 (9.6%)	16 (15.5%)	55 (10.8%)
55–64	10 (32.3%)	29 (24.6%)	19 (20.6%)	22 (31.4%)	80(25.7%)
≥ 65	17 (27.4%)	26 (30.9%)	34 (45.9%)	19 (40.4%)	96 (35.9%)
**Isolated diastolic hypertension n = 72**					
Overall	15 (5.2%)	34 (6.0%)	18 (3.6%)	5 (1.4%)	72 (4.2%)
35–44	7 (5.4%)	11 (4.5%)	9 (3.7%)	1 (0.51%)	28 (3.4%)
45–54	4 (4.5%)	15 (9.0%)	3 (2.2%)	0 (0.00%)	22 (4.6%)
55–64	3 (12.5%)	8 (8.2%)	4 (5.2%)	2 (4.0%)	17 (6.9%)
≥ 65	1 (2.2%)	0 (0.00%)	2 (4.8%)	2 (6.7%)	5 (2.8%)
**Previous history of hypertension = 99**					
Overall	24 (6.8%)	51 (7.3%)	6 (0.95%)	18 (3.9%)	99 (4.6%)
35–44	4 (2.8%)	7 (2.6%)	1 (0.4%)	6 (2.8%)	18 (2.0%)
45–54	9 (9.1%)	14 (7.0%)	1 (0.6%)	5 (4.7%)	29 (5.1%)
55–64	3 (7.7%)	15 (10.8%)	0 (0.00%)	4 (4.8%)	22 (12.3%)
≥ 65	8 (11.3%)	15 (15.9%)	4 (4.6%)	3(5.4%)	30 (9.7%)

**Variable Specification:** *HBP*:*-High blood pressure*: *(systolic blood pressure ≥140mmHg and/ or diastolic blood pressure ≥ 90 mmHg or taking antihypertensive medication)*. *Isolated systolic hypertension*: *systolic blood pressure ≥ 140 mmHg and diastolic blood pressure <90 mmHg*, *Isolated diastolic hypertension*: *systolic blood pressure < 140 mmHg and diastolic blood pressure ≥ 90 mmHg; Previous history of hypertension (taking antihypertensive medication)*.

### Prevalence of hypertension

**Fig 1:**
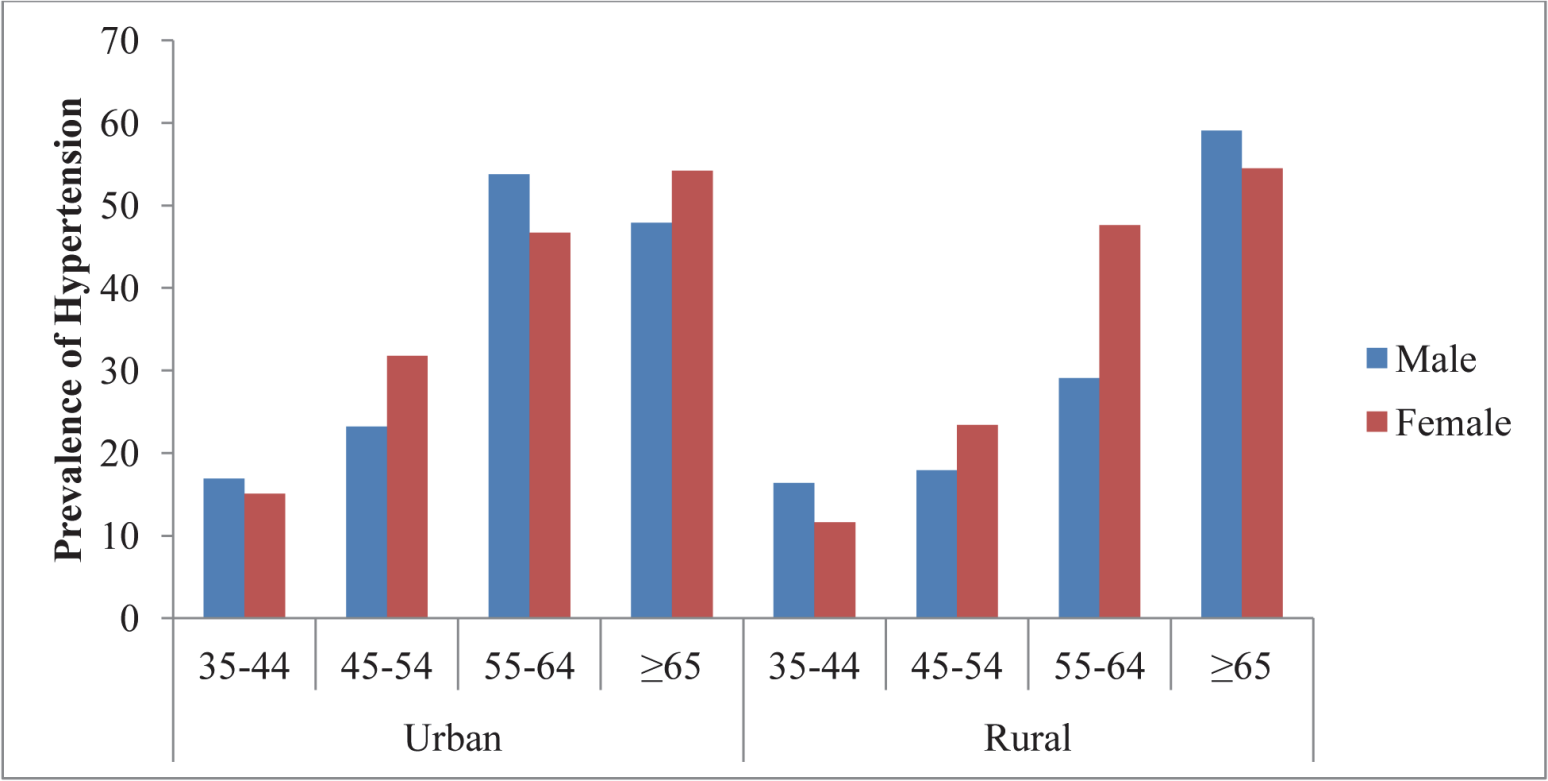
Prevalence of high blood pressure by socio-demographic characteristics and residence of the study population among adults age 35 years and older, northwest Ethiopia, (2012).

The overall prevalence of hypertension was found to be 27.9% [95% CI: (26.0, 29.8)]. The proportion was 30.7% [95% CI: (27.9, 33.4)] in urban study subjects and 25.3% [95%CI: (22.7, 27.9)] in rural study subjects. The sex specific prevalence of hypertension was 29.3% [95%CI: (26.7, 31.9)] for women and 23.3% [95%CI: (23.5, 29.1)] for men (Fig 1).

The prevalence of known hypertensive patients taking antihypertensive medication was 99 (4.6%). The prevalence of Isolated systolic hypertension was 294 (13.7%) [95%CI: (12.3, 15.2)]. The proportion which was 166(15.2%) in rural areas was slightly higher for urban areas which was 128(12.2%). It was 133(13.6%) for male and 161(13.9%) for female. The proportion of isolated systolic blood pressure increased with increasing age. The prevalence of Isolated diastolic hypertension was found to be 72 (3.4%) [95%CI: (2.6, 4.1)]. And this was higher 49 (4.6%) [95%CI: (3.4, 5.9)] among urban dwellers than 23 (2.1%) [95%CI: (1.2, 2.9)] among rural dwellers. Of this, 33 (45.8%) were male and 39 (54.2%) female (Table 1). Among the 598 participants identified as hypertensive, 241 (40.3%) never had BP measurements, and 99 (4.6%) had known hypertension and were on treatment. Of the patients who were on antihypertensive treatment, 50.5% had controlled hypertension at the time of the study (BP < 140/90 mmHg).

The proportion of systolic and diastolic hypertension in this subgroup of adults was 133(6.2%), 62 (46.6%) for males and 71 (53.4%) for females. Among all subjects with hypertension, 357 (59.7%) had no previous measurement of blood pressure and were not aware of their hypertension until the time of the survey.

### Factors

After adjusting for a number of important covariates, the multivariable logistic regression analysis showed that when a person got older by one year, the odds of hypertension occurrence increased by 6% (AOR = 1.06; 1.05, 1.07), (as age increased the prevalence of hypertension increased). Currently alcohol consumption (AOR = 1.71; 1.24, 2.36), was significantly associated with hypertension and on average a one unit increase in BMI (kg/m^2^) led an increase of 7% on the occurrence of hypertension, (AOR = 1.07; 1.04, 1.10). Raised fasting glucose had a marginal significance (AOR = 1.01; 1.001, 1.01) (Table 2).

The multivariable logistic regression analysis showed that living in urban areas, and alcohol consumption was significantly associated with Isolated Diastolic Hypertension (IDH). Urban dwellers were more likely to be IDH compared to rural dwellers (AOR = 3.25; 1.67, 6.29). Current alcohol users were more likely to be IDH compared to participants reporting to have never consumed alcohol (AOR = 2.30; 1.23, 4.28), while old age, and current alcohol use were significantly associated with Isolated systolic hypertension (ISH). When a person got older by one year, the odds of ISH occurrence increased by 6% (AOR = 1.06; 1.04, 1.07), and current alcohol users were more likely to be ISH compared to participants reporting no use (AOR = 2.05; 1.36, 3.09) (Table 3).

**Table 3:** Multivariable analysis of factors associated with hypertension among urban and rural residence of Gondar, North West Ethiopia (2012).

Variable	Study population	Hypertension	Adjusted OR [95%CI]
	n (%)	n %	
**Age in year**	N = 2141	598 (27.9%)	1.06 [1.05,1.07] *
**Waist circumference**			1.01 [0.99,1.02]
**Fasting blood glucose mg/dl**			1.01 [1.001,1.01] *
**BMI Kg/m2**			1.07 [1.04,1.10] *
**Sex**			
Male	981 (45.8%)	258 (26.3%)	1
Female	1160 (54.2%)	340 (29.3%)	1.20 [0.97, 1.50]
**Residence**			
Rural	1091 (51.0%)	276 (25.3%)	1
Urban	1.050 (49.0%)	322 (30.7%)	1.25 [0.91, 1.71]
**Have you currently consumed alcohol**			
No	791 (37.0%)	228 (28.8%)	1
Yes	1350 (63.0%)	370 (27.5%)	1.71 [1.24, 2.36]*
**Smoking**			
No	2,116 (98.8%)	594 (28.1%)	1
Yes	25 (1.2%)	4 (16.7%)	1.43 [0.47,4.31]
**At least Moderate Physically active**			
Yes	1,657 (77.4%)	428 (25.8%)	1
No	484 (22.6%)	170 (35.2%)	1.20 [0.91,1.57]

**Independent variables in this model were:** Age, sex, residence, waist circumference, fasting blood glucose mg/dl, alcohol consumption, BMI Kg/m^2^, and doing moderate physical activity.

## Discussion

In this population-based cross-sectional study, we were able to identify a high prevalence of hypertension in a rural population of northwest Ethiopia. We also found that a large proportion of the hypertension was undiagnosed and thus untreated. The associated factors included obesity, old age, alcohol consumption, and increasing waist circumference.

The urban prevalence of hypertension in our finding was similar with earlier reports from urban Gonder(28%) and Addis Ababa (31.5%) male and (28.9%) female [7,17,18]. This is higher than the recent prevalence report for the employees of the Commercial Bank of Ethiopia in Addis Ababa, which could be due to the fact that the mean age of our study population was higher than that the employees of CBE [8]. Although this silent epidemic constitutes a big burden to the country, it is not getting enough, preventive and controlling intervention from the health care system.

The prevalence of hypertension in the rural area was higher than the 2007 report from a rural and semi-urban community of Butajira DHSS which was (12.3%) for men and (8.2%) for women [17]. These findings seem to suggest that the protective effects against hypertension in the studied rural areas are diminishing in our time. One possible explanation for the increasing magnitude of hypertension trends in rural settings may be the changes in lifestyles with the adoption of urban ways of life. Moreover, there was variation of age distribution among the two study populations [19]. Furthermore, the increasing prevalence in our report showed that the burden of the disease was increasing from time to time and alarmingly increasing among the rural people who were losing their protective lifestyle and dietary habits.

Among the subjects who were identified as having hypertension, only 16.4% were on antihypertensive treatment; the rest (83.4%) were not diagnosed and treated. This finding was in line with that of study done on urban dwellers of Addis Ababa which was (64.8%), but much higher than an earlier report by a study on the urban population of Gondar (37%) [18] [7]. The finding is alarming in that there is a huge hidden burden of undiagnosed hypertension both in the urban and rural populations and requires urgent attention. If we include those with pre-hypertension, the magnitude of the problem will be much higher. What makes it even worse is the fact that among those who are on treatment, only about 50% had controlled the hypertension. It’s an established fact that hypertension is an important modifiable risk factor for cardiovascular, cerbrovascular, and renal diseases. Therefore, the finding calls for a more comprehensive intervention against hypertension in terms of prevention, screening, and proper management of the disease.

In our findings, increasing fasting blood glucose was the major independent variable associated with hypertension. The overall prevalence of IFG in our study was lower than the Addis Ababa report (21.6%) and similar with that of a study done in Morocco, 5.5% [8,20]. Moreover, the computed prevalence of IFG differs significantly with areas of residence and age. The rate increased modestly with age and highly among urban dwellers. Similarly, a study conducted in South Africa showed the prevalence of IFG increased with age, with peak prevalence in the oldest age-group. [21–23]. Impaired Fasting Glucose (IFG) is a predictor of the incidence of Type 2 diabetes and hypertension [23,24]. Epidemiological studies and path-physiological mechanisms provide evidence to the co-existence of hypertension, IFG, and abdominal obesity, possibly pointing towards a common genetic and environmental factor promoting the risk of CVD [25,26]. Similarly, there is a strong association between abdominal obesity, rise in FBG, and hypertension which was also observed in our finding [27].Therefore, the persistent high prevalence of IFG may be a possible predictor of a further increase in hypertension in this population in the years to come [28].

With increasing age, there will be increasing stiffness of the aorta and arterial walls which contribute to the observed high prevalence of hypertension in the older group. In the current study, increasing age was an important risk factor for hypertension. Overall, the age-standardized prevalence rate was found to be significantly associated with hypertension, with much higher prevalence noted in the older age compared to the younger. The increase in blood pressure with age was consistent with many studies, more importantly with previous reports from studies in Ethiopia and Ghana [7,29]. Increased stiffness of the aorta and the arterial with age could contribute to the observed high prevalence in this group.

In our finding, obesity was the major independent variable associated with hypertension detected using both BMI and waist circumference for the determination of obesity. As we have seen, the forces promoting sedentary behavior have grown substantially over the last few decades. Urbanization is associated with changes in dietary habits and with reduced physical activity that lead to obesity. Such changes of lifestyle and dietary habits contribute to the excess prevalence of abdominal obesity in urban areas which eventually result in the increased prevalence of hypertension [[13]. Urbanization is associated with changes in dietary habits and exercises that lead to obesity which have been implicated as contributing factors in the development of high blood pressure.

Alcohol consumption was significantly associated with high blood pressure in the study population. About 81% of the rural study population reported regular alcohol consumption. Previous research indicated that heavy alcohol consumption was a risk factor for high blood pressure [30–32]].

One of the limitations of this study was the inclusion of only adults above the age of 35 years, which could contribute to an overestimation of the overall prevalence of hypertension in the general population, while the majority of Ethiopians are in the younger age group. We opted to survey older age group to get more cases to be able to look into the risk factors, which obviously increase the prevalence of hypertension compared to younger adults. Lack of details on exposures, such as salt intake, renal function test, and use of contraceptive drug is the other limitation of the study. Also some of the information was based on self-reports and is subject to recall bias. Variations in the blood pressure measurement methodology, age range of the study participants, and the variation in the residence of the populations make direct comparisons of studies difficult.

## Conclusion

The prevalence of hypertension in the rural area is considerably higher in northwest Ethiopian. Along with the increased prevalence of IFG, there is a high risk of developing cardiovascular diseases. Increasing age, raised fasting blood glucose, alcohol consumption, obesity and high waist circumference were significantly associated with hypertension. The implications of the findings are important since nearly 84% of the population of Ethiopia still lives in the rural area, and if corrective action is not taken, there will be unbearble health consequences. Therefore, the health system needs to develop strategies to increase the reach of relevant screening and diagnostic services to both rural and urban populations. Interventions will require a reorientation of awareness so that primary healthcare services go towards the primary prevention and management of the needs of older adults. These interventions may include weight loss, dietary sodium reduction, moderation in alcohol consumption, and increased physical activity,
